# New Bio-Based Polymer Sorbents out of Terpene Compounds or Vegetable Oils: Synthesis, Properties, Analysis of Sorption Processes

**DOI:** 10.3390/polym14245389

**Published:** 2022-12-09

**Authors:** Magdalena Sobiesiak

**Affiliations:** Department of Polymer Chemistry, Faculty of Chemistry, Institute of Chemical Sciences, Maria Curie Sklodowska University in Lublin, Gliniana 33, 20-614 Lublin, Poland; magdalena.sobiesiak@umcs.pl

**Keywords:** porous polymers, bio-based polymers, terpenes and terpenoids, vegetable oils, SPE, phenols, ibuprofen, paracetamol, aspirin, salicylic acid

## Abstract

This research presents a synthesis and characterization of new bio-based polymer sorbents. Natural origin substances such as terpenes (citral, limonene, and pinene) or vegetable oils (argan, linseed, and rapeseed oils) were used as monomers, and divinylbenzene was applied as the cross-linker. The newly prepared polymers were characterized by means of ATR-FTIR, TG/DTG and titration methods (acid and iodine values), and N_2_ physisorption experiments. Tests of sorption ability were carried out by a dynamic solid phase extraction method using a mixture of four phenols or single-component pharmaceutical solutions (salicylic acid, aspirin, ibuprofen, paracetamol, and ampicillin). The performed studies revealed that the terpene-based polymers possessed better-developed porous structures (420–500 m^2^/g) with more uniform pores than oil-based ones. However, the surface of the oil-based sorbents was more acidic in nature. The sorption tests showed that both the porosity and acidity of the surface significantly influenced the sorption. Recoveries of up to 90% were obtained for 2,4 dichlorophenol from C-DVB, L-DVB, and Ro-DVB. The lowest affinity to the polymers exhibited phenol (5–45%), aspirin (1–7%), and ampicillin (1–7%). A 70% recovery was achieved for ibuprofen from C-DVB. In-depth data analysis allowed the influence of various factors on the sorption process of test compounds of the studied polymers to be elucidated.

## 1. Introduction

Many naturally occurring organic substances are of an unsaturated chemical nature due to the presence of one or more double bonds present in their molecules, which not only gives them specific chemical properties, it also affects their physical features, such as the state of aggregation, melting, and boiling points.

The vast majority of unsaturated organic compounds are substances of plant origin. Depending on the functions performed in the plant organism, they can occur in different parts of the organism (e.g., leaves, seeds), or be a component of essential oils, resins, or saps. Terpenes and (glycero)lipids (vegetable fats and oils), inter alia, are representative of this group of compounds.

Terpenes are natural hydrocarbons built of isoprene units (C_5_H_8_), which when joined together, can form chains or rings. Depending on the number of isoprene units linked together, terpenes are classified into monoterpenes (C_10_H_16_—two isoprene units), sesquiterpenes (C_15_H_24_—three units), diterpenes (C_20_H_32_—four), and polyterpenes ((C_5_H_8_)_n_—many). For this reason, compounds belonging to each of these groups have exactly the same chemical formulas, although their structures are different due to isomerism. Terpenes are highly flammable, from colorless to yellow liquids of a non-polar nature [1,2].

Being natural derivatives of terpenes, terpenoids are the second large group of compounds that are very diverse in terms of their chemical structure. Terpenoids are composed of isoprene units to which various functional groups are attached; often there are oxygen moieties, such as hydroxyl, aldehyde, ketone, or oxygen carboxyl groups. For this reason, terpenoids have very similar physical properties to terpenes; however, they are more polar. Higher polarity results in slightly better water solubility and a little less volatility in comparison to terpenes [1].

The biological activity of terpenes and terpenoids allows them to be used as ingredients in natural medicines and pharmaceuticals, as well as within plant protection agents (natural pesticides). Their characteristic flavors and pleasant fragrances provide them with wide and common applications in the food, cosmetics, and perfumery industries. Due to their ability to dissolve various substances, terpene compounds are utilized as degreasing and cleaning products and organic solvents. Chemical reactivity is the reason for terpenes and their derivatives to be applied as reagents in chemical syntheses. They can also constitute a feedstock for the chemical industry, e.g., in the preparation of adhesives, varnishes, inks, paints, etc. [1,3].

Terpenes and terpenoids have also been used in the synthesis of polymeric materials as monomers that meet the requirements of green and sustainable chemistry [4]. Natural features of terpenes, such as lipophilicity, liquid crystalinity, bioactivity, and biodegradability, make the polymers produced out of them useful in many applications.

In order to obtain polyterpenes, chain-growth and step-growth polymerizations with appropriately selected catalysts have been designed [3,5,6]. Polymerizations of terpene-based monomers have also been widely used. In this approach, terpenes are chemically modified to give new monomers with unique properties. Succinic or maleic anhydrides [3,6,7], acid chlorides, or methyl methacrylate [3] are examples of popular compounds, the use of which obtains new terpene-based polyesters. In turn, the utilization of citric aminoamide [8] enables the synthesis of polyurethanes. There are also known methods to obtain polymers with pendent terpenes entities using, for example, graft reactions. When attached to the backbone of another polymer, to a greater extent, such terpenes preserve some of their properties, such as biological activity [3].

Intensive scientific works carried out in this field have resulted in many new monomers and polymers, which have opened up great potential in terms of the desired material properties, such as high chemical resistance [7] or recyclability [6].

Vegetable oils composed mainly of triglycerides are another interesting group of unsaturated compounds. These are relatively cheap and easy-to-obtain substances. The most common and obvious use of vegetable fats and oils is in the consumption food industries. However, their applications in other fields are also well-known. These result from the specific properties of vegetable oils, which are a consequence of the unique chemical composition of glycerolipides (Table 1).

Argan oil, known as the “liquid gold of Morocco”, is famous for its cosmetic applications [9,11,12,13], but it also can be used as a costabilizer in miniemulsion polymerization [14]. Linseed oil is utilized in pharmaceutical and cosmetic products (e.g., soaps), but also as an industrial raw material for the production of wood preservatives (especially those with hydrophobic properties). Various preparations based on this oil are used as binders and media in painting or the restoration of works of art and antiques [15]. Rapeseed oil can be applied as a fuel or admixture with diesel oil; it is also a raw material for the production of biofuels such as biodiesel [16,17,18].

In the era of dwindling natural resources, new sources of chemical reagents are sought that will be able to partially or completely replace the current petroleum-derived substrates. Hence, there is a constant increase in the use of unsaturated compounds of natural origin [10,19]. In particular, many industrial applications do not require compounds of the highest quality, as is demanded in the food or pharmaceutical industries. Indeed, a desirable direction is the search for management methods:

- Post-production waste, (e.g., post-extraction residues),
- The utilization of substrates that are unsuitable for specific purposes, e.g., pharmaceutical production due to lower quality or food purposes because of expiring shelf life,
- Exploiting matter that is not suitable for further use (e.g., post-frying oils). Often the final stage of using such substances is adding them to fuels for energy retrieval purposes. An alternative may be the production of polymeric materials.

According to the typical chain-growth polymerization mechanism, natural triglycerides react slowly; therefore, in order to increase their reactivity, methods based on initial chemical modification have been proposed, e.g. oxidation of the double bond to the epoxide which, after hydrolysis, gives diols used in the preparation of polyurethanes. The epoxidized intermediate may also condense with acrylic/methacrylic acid, ester, or amides to form new monomers suitable for thermoset plastic production [19]. They can also be copolymerized with, for example, styrene, which significantly expands the possibilities of obtaining polymers with various properties, from rigid and hard cross-links to soft rubber-like ones. Fatty acids after reaction with diols and dithiols produce polymers intended for medical applications, such as drug delivery systems, tissue engineering, or temporary implants [5].

As described above, many new non-porous polymers based on terpenes or vegetable oils have been obtained and found useful for practical applications [3,5]. However, to the best of this author’s knowledge, there is still little space in the literature dedicated to the topic of using such substances in the synthesis of porous polymers for purification and separation processes [20]. This opens up another perspective for completely new applications of terpene compounds and vegetable oils as substrates for the synthesis of porous materials.

Polymer sorbents are widely used in separation and purification techniques due to their many advantages, such as well-developed surface area and thermal resistance, and chemical resistance, including not only organic solvents but also solutions with low or high pH. An additional advantage is also the great possibility of adjusting the porosity or chemical structure of the surface to specific needs. For this reason, many synthetic polymers, such as polystyrene-divinylbenzen, N-vinylpyrrolidone-divinylbenzene, and methacrylate-divinylbenzene, polyamides, are in common commercial use as column and cartridge fillers in chromatography and solid phase extraction techniques, for the determination of analytes from various types of matrices, such as natural water, sewage, biological material (blood, urine, etc.), or food and beverages [21,22,23].

Among the compounds that have been subjected to constant monitoring for many years are phenols and their derivatives [24,25]. These compounds, found as anthropological origin impurities in drinking water, give water an unpleasant characteristic smell and spoil its taste. In addition, di- and tri-substituted chlorophenols can bioaccumulate and pose a serious threat to aquatic organisms, and consequently also to humans [26].

Drugs are another group of compounds of analytical interest. In this case, the research has developed in many directions and includes, for example:

- Testing biological samples to determine the presence or bioavailability of drugs, or the presence of their metabolites when determining the tracks and speed of their metabolism in the body as well as in drug delivery systems [27,28],
- Isolating drugs from pills during quality control or disposal [29],
- Testing food products, water, and sewage for the presence of drugs or their derivatives [30,31,32,33,34].

The aim of this work is to present the method of using unsaturated substances of natural origin in the synthesis of porous polymer microspheres. These newly synthesized polymers can be considered as alternative bio-based, sustainable, or semi-green sorbents, in the synthesis of which some of the petroleum-derived monomers, for example, very popular styrene, have been replaced with substances of natural origin such as terpenes or vegetable oils.

The obtained materials were characterized by infrared spectroscopy and chemical analysis to confirm the assumed course of the reaction. Porous structure parameters were defined with low-temperature nitrogen sorption measurements. The thermal properties were studied using thermogravimetry (TG) and differential thermogravimetry (DTG) techniques. In order to evaluate the potential practical applications of the obtained polymers, preliminary studies of sorption properties using the method of dynamic solid phase extraction were carried out. In the first stage of the research, phenol and three of its chlorinated derivatives were used as test compounds. The obtained results were satisfactory with recoveries up to 90% (for 2,4 dichlorophenol) achieved for three polymers: C-DVB, L-DVB, and Ro-DVB. This prompted tests for other biologically active compounds, such as non-steroidal anti-inflammatory drugs (aspirin, ibuprofen, paracetamol), salicylic acid, and antibiotic (ampicillin). The drugs containing strongly acidic characters such as ampicillin, aspirin, and salicylic acid had the lowest affinity to the polymers and their recoveries ranged from 1% to around 37%. The highest recovery reached 70% and was obtained for ibuprofen from C-DVB.

The obtained results showed the new materials could be potentially used as reusable sorbents for slightly acidic aromatic compounds, including pharmaceuticals such as ibuprofen or paracetamol. Additionally, in-depth analysis of the collected data allowed for the assessment of the influence of various factors on the process of uptake of test compounds from aqueous solutions.

## 2. Materials and Methods

### 2.1. Chemicals, Reagents and Solvents

65% divinylbenzene (DVB), methanol (MeOH), 2-chlorophenol (2-ChP), 2,4-dichlorophenol (2,4-DChP), and 2,4,6-trichlorophenol (2,4,6-TChP), 1,4-dioxane and 2-hydroxybenzoic acid (salicylic acid), acetylsalicylic acid (aspirin), (S)-(+)-2-(4-Isobutylphenyl)propionic acid (ibuprofen), 4-acetamidophenol (paracetamol), ampicillin, bromine, and starch were purchased from Merck (Darmstadt, Germany). Hexane, acetone, toluene, and phenol (P), poly(vinyl alcohol) (PVAL), 99.8% acetic acid, iodine, NaOH, KI, Na_2_S_2_O_3_, were from POCh (Gliwice, Poland). Linseed oil (Lo) and rapeseed oil (Ro), both cooking grade, and argan oil (Ao) cosmetical grade 100% (cold pressed), were purchased from local suppliers. 97% (R)-(+)limonene (L) was from Aldrich (Steinheim, Germany). 96% cis/trans citral (C), 97% (1R)-(+)-alfa-pinene (P) was from SAFC, and 98% α,α’-Azoiso-bis-butyronitrile (AIBN) was from Fluka (Busch, Switzerland).

### 2.2. Synthesis of Bio-Based Porous Polymeric Microspheres

All the polymeric materials were prepared by suspension polymerization in accordance with previously developed procedures [35,36]. 3 g of PVAL was mixed with 120 mL of distilled water in a three-necked rounded flask equipped with a condenser, a mechanical stirrer, and a thermometer. The suspension stabilizer solution was heated at 80 °C and stirred until the PVAL was completely dissolved. In a separate vessel, a reaction mixture was prepared of: renewable ingredients of natural origin (C, P, L, Ro or Lo)—5 g, cross-linking monomer (DVB)—5 g, 15 mL of toluene as an effective pore-forming agent [37] and 0.150 g of AIBN—radical initiator. The mixture was carefully heated to 80 °C and immediately poured into a reaction flask containing the hot and continuously stirred PVAL solution (ca. 250 rpm). Under these conditions, the polymerization process was carried out for 20 h. After this time, the obtained polymer product was filtered off and washed in an ultrasonic bath with several portions of methanol to completely remove any remaining reagents from the porous structure. The purified material was gently dried at room temperature. Material designations are given in Table 2

### 2.3. Methods of Characterization

Infrared spectra with Fourier Transform were collected in Attenuated Total Reflectance mode (ATR-FTIR) using a FTIR spectrophotometer TENSOR 27 (Bruker, Ettlingen, Germany). The spectra were recorded in the frequency range of 4000 to 600 cm^−1^ and the resolution of the apparatus was 4 cm^−1^. Each measurement included 32 scans per spectrum.

Additionally, chemical studies were performed to determine characteristic values, such as acid (AV) and iodine values (IV). Quantification was carried out through back titration methods. Details are given in Appendix A.

Porous structure parameters of the synthesized polymers were determined by low-temperature nitrogen sorption experiments with an ASAP 2405 analyzer (Accelerated Surface Area and Porosimetry system, Micromeritics Inc., Norcross, GA, USA). Before analysis, the samples were outgassed at 110 °C for 1 h. The isotherms were measured at 196 °C. The specific surface area (S_BET_) was calculated using the standard BET method assuming N_2_ molecular cross-sectional area 0.162 nm. The total pore volume (V_tot_) was determined as the volume of liquid nitrogen at a relative pressure (p/p^0^) of 0.99. The pore size distribution (PSD) in the range of 1.7–300 nm and average pore diameter (D_BJH_) were calculated according to BJH procedure from the desorption branch of the isotherm.

SEM images were taken with the use of a high-resolution scanning electron-ion microscope Quanta 3D FEG (Hillsboro, OR, USA) by FEI.

TG/DTG thermograms were obtained through an STA 449 F1 Jupiter thermal analyzer (Netzsch, Selb, Germany). Around 7 mg of a sample was weighed into an Al_2_O_3_ crucible and analyzed under helium atmosphere (40 mL min^−1^) in the temperature range of 30 to 800 °C with standard heating rate of 10 K/min.

Sorption property tests were performed by dynamic solid phase extraction (SPE) methods using polypropylene cartridges filled with 100 mg of studied polymer and protected with porous Teflon frits. Before use, each cartridge was conditioned with 10 mL of methanol and 5 mL of distilled water. Next, aqueous solutions of test compound were passed through the cartridges at a mean flow rate of 3 mL/min.

First, tests with a mixture of phenolic compounds were conducted. The solution consisted of 2 mg/L: phenol, 2-chlorophenol (2-ChP), 2,4-dichlorophenol (2,4-DChP), and 2,4,6-trichlorophenol (2,4,6-TChP). After sorption, the cartridge was rinsed with 1 mL of water and then dried for 5 min. Next, the elution with an appropriate amount of methanol was carried out. The amount of methanol was determined by the sample volume in such a way to obtain a 50-fold concentration, e.g., if the sample volume was 100 mL, 2 mL methanol was used for the elution. Finally, the cartridge was regenerated in the same manner as in the conditioning step.

Subsequent sorption tests included the recovery determination of chosen pharmaceuticals (salicylic acid, aspirin, ibuprofen, paracetamol, ampicillin) from aqueous solutions. For this purpose, separate pharmaceutical solutions with a concentration of 0.2 mg/L and 100 mL volume were prepared. The adsorbed compounds were eluted with 2 mL of methanol.

Concentrations of desorbed compounds in the obtained eluates were determined using HPLC system Waters 2690 Alliance (Waters, Milford, MA, USA) with UV detector; a detailed description has been given in previous works [36,38]. For phenolic eluates, the analysis parameters were as follows: mobile phase: methanol-water (60:40, *v*/*v*), a flow rate of 1 mL min^−1^, and detection at λ = 210 nm; composition of the mobile phase for pharmaceutical eluates: methanol-water (80:20, *v*/*v*), a flow rate of 1 mL/min, and detection performed at λ = 222 nm. The collected results are presented in the form of graphs showing the percentage recovery value as a function of the sample volume.

## 3. Results and Discussion

### 3.1. Infrared Spectroscopic and Chemical Analysis

New porous polymer microspheres containing the natural component were synthesized by the suspension method. In this approach, a composition of monomers, the initiator, and porogens is poured into the protective colloid solution to allow small droplets to be formed in which the polymerization process takes place. An advantage of this technique is that by proper selection of reactants and synthesis parameters, it is possible to obtain a polymer product with desirable properties, such as the size of polymer beads or their porosity.

**Figure 1:**
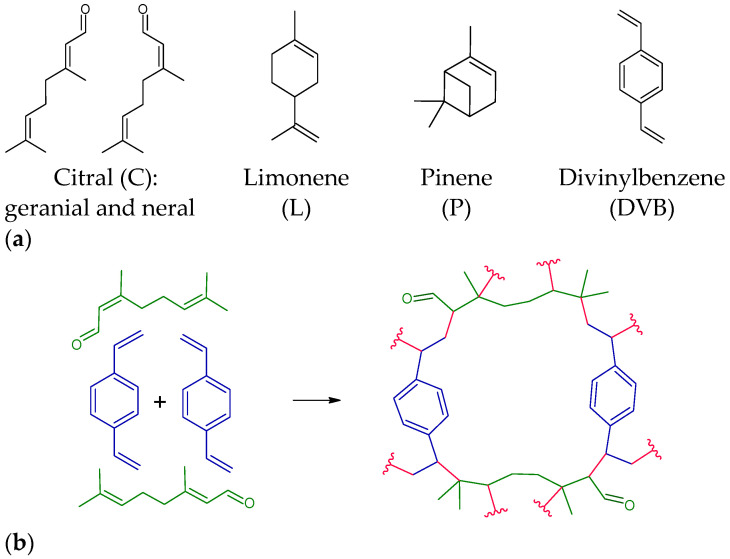
(**a**) Chemical formulas of terpenes/terpenoids monomers and divinylbenzene. (**b**) A hypothetical scheme of polymerization and formation of porosity. Monomers of citral (geranial and neral) are green, and divinylbenzene are blue. In the polymeric structure, the fragments from the appropriate monomers are marked with relevant colors, and the newly formed bonds are marked in red.

Figure 1a shows the molecular formulas of the terpene monomers: limonene and pinene, and the terpenoid monomer—citral. Citral is the equimolar mixture of two stereoisomers geranial and neral. These unsaturated organic compounds are able to react with divinylbenzene to form macromolecular chains. These chains are entangled with one another and some connect with others to form a three-dimensional spatial network. A simplified scheme of a hypothetical reaction between citral (geranial and neral) and divinylbenzene is presented in Figure 1b. In a cross-linked polymeric structure, the bonded chains create spaces of limited size, which determine the porosity.

The reaction is similar in the case of triglycerides contained in vegetable oils. The esters of unsaturated fatty acid copolymerize with divinylbenzene give polymeric products. In the next stage, hydrolysis or transesterification can be performed to modify their chemical and physical properties.

**Figure 2:**
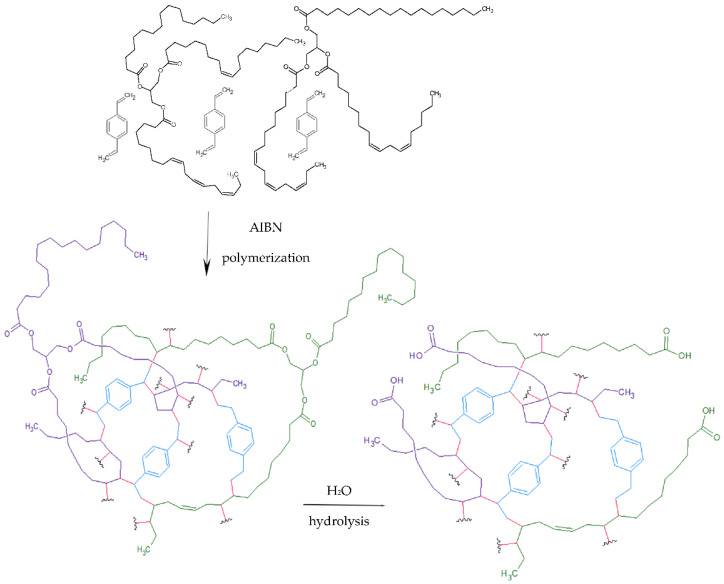
General scheme of polymerization of fatty esters with divinylbenzene followed by hydrolysis.

A possible course of polymerization between divinylbenzene and molecules of triglycerides (which may be included in linseed, rapeseed, and argan oils) is schematically presented in Figure 2. To not specify any oil, the molecules of triglycerides were shown as esters of glycerol and stearic, oleic, linoleic, and linolenic acids.

**Figure 3:**
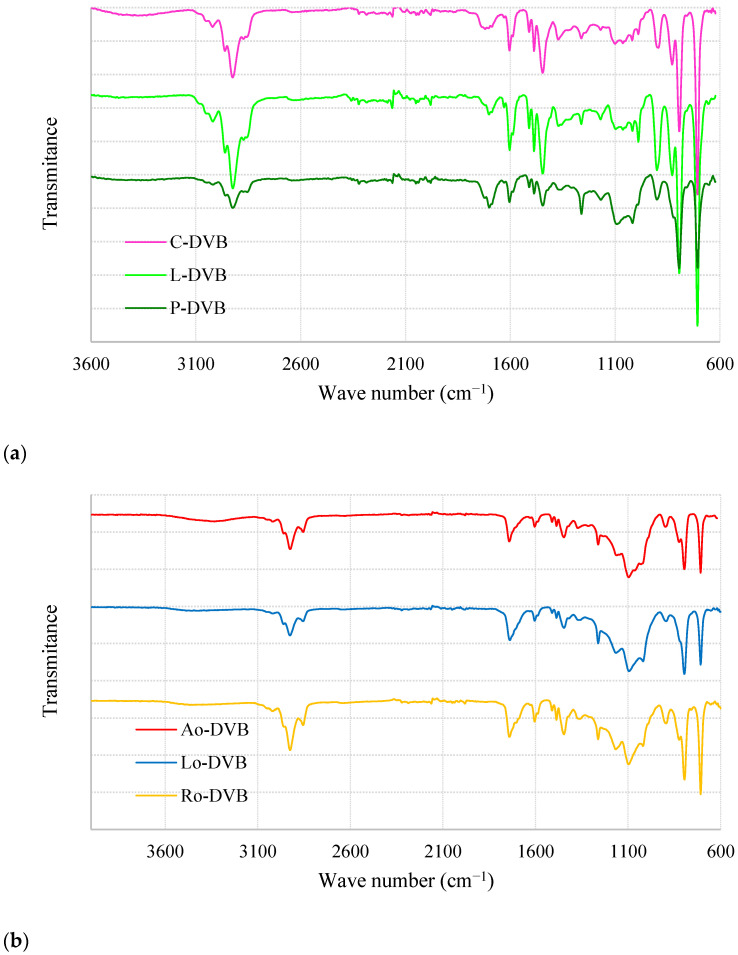
ATR-FTIR spectrograms of (**a**) C-DVB, L-DVB, and L-DVB and (**b**) Ao-DVB, Lo-DVB, and Ro-DVB.

To control and confirm the preparation of the polymers possessing designed chemical composition infrared spectra were analyzed. Figure 3 presents the collected ATR-FTIR spectra grouped into the terpene-based materials (a) and vegetable oil-based ones (b).

In all spectra, bands characteristic of DVB species were observed. The two strongest at 707 and 793 cm^−1^, and two less intense at 829, 899 cm^−1^, resulted from out-of-plane deformation of the aromatic ring. Whereas in the plane ring, deformation bands (=C–H) were observed at about 1095, 1070, and 989 cm^−1^. Stretching vibrations of aromatic system absorb in the range of 1625 to 1430 cm^−1^, their in-tensities are medium. The last range of characteristic vibration covered 3000–2800 cm^−1^, where symmetric and asymmetric alkane C–H stretching was observed. These bands confirmed the presence of aliphatic chains forming a spatial polymeric network [39,40].

**Figure A1:**
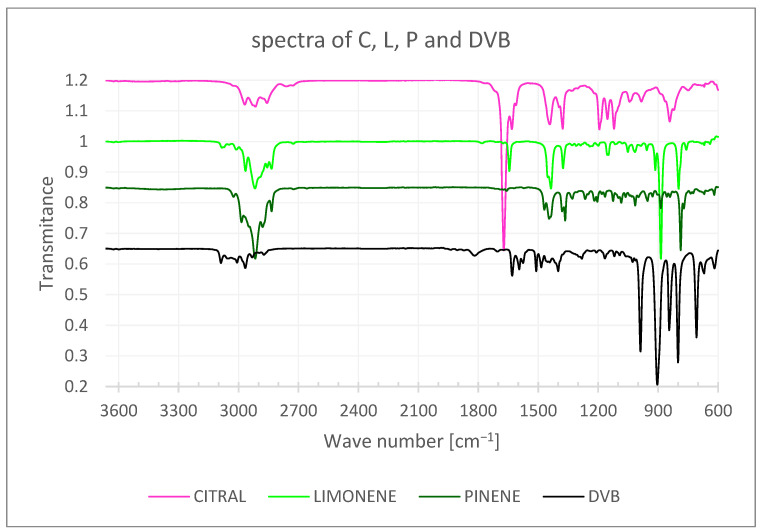
Spectra of the terpenes and DVB.

**Figure A2:**
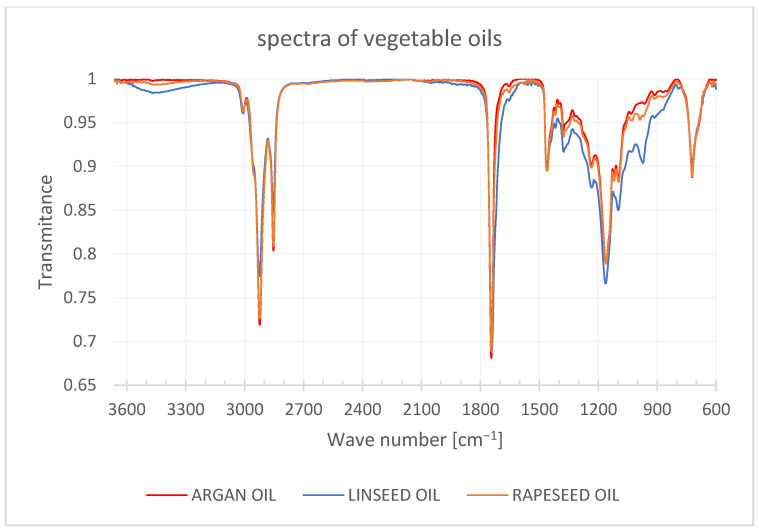
Spectra of the vegetable oils.

**Figure A3:**
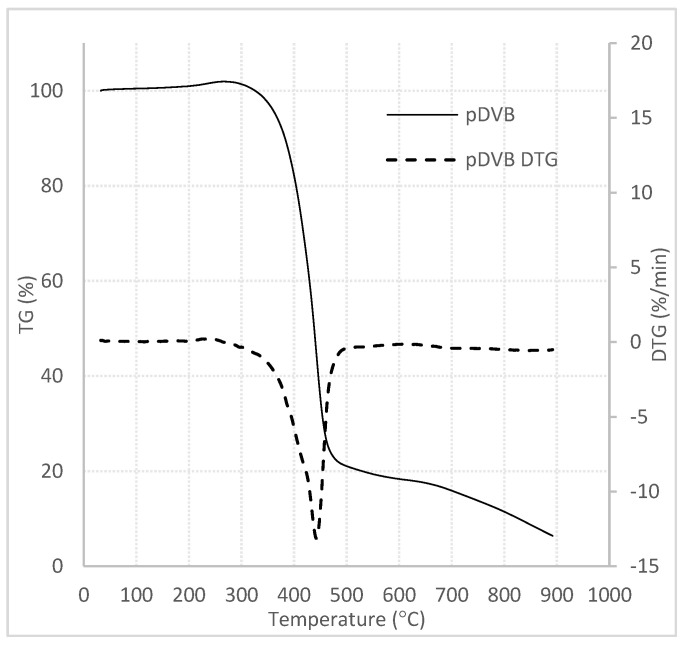
TG/DTG curves of pDVB.

Additionally, each spectrum contained bands characteristic of functionalities or structure of the second monomer, which proved its presence in the polymer. The spectra of the terpene monomers and vegetable oils can be found in Appendix A (Figure A1, Figure A2 and Figure A3 and Table A1).

Typical vibration of the aldehyde group was observed in the range of 1740–1720 (for saturated), 1715–1695 (for aromatic), and 1705–1680 cm^−1^ (for α,β-unsaturated ones). In the case of C-DVB (Figure 3a), aldehyde vibrations were visible as a broad band in the range of 1750–1660 cm^−1^. The shape of the band suggested that various vibrations of the carbonyl groups contributed to its formation, including derivatives like ketones. Another band situated at 1095 cm^−1^ resulted from in-plane deformation of the C–C=O bond. A broad and flattened band in the range of 3200–3500 cm^−1^ and another of medium intensity at 1017 cm^−1^ indicated the presence of hydroxyl groups (which can arise as the result of keto-enol tautomerism or a side reaction of hemiacetal formation [41,42,43]).

The most characteristic element of the limonene structure was its six-membered aliphatic ring. Its vibrations in pure limonene were observed as three medium-intensity bands in the range of 970–1060 cm^−1^. Two (at 989 and 1015 cm^−1^) were visible in the spectrogram of L-DVB (Figure 3a). A significant reduction in intensity, or even the absence of bands caused by C=C at 1645 cm^−1^ in cyclohexene ring and vinylidene moieties of limonene, indicates the considerable degree of conversion of these bonds during the polymerization process [44].

Limonene and pinene possess the same basic structural fragment; for this reason, the spectrum of P-DVB (Figure 3a) bands at 1016 and 990 cm^−1^ was also present. However, the ratio of their intensities was completely different. In the case of P-DVB, the former was stronger, while the latter was hardly visible. The vibrations of the four-membered ring had their strongest band at 1445 cm^−1^ but this wave number was also characteristic of aromatic ring stretching and methylene C–H scission vibrations. Carbon-carbon skeletal vibration of >C(CH_3_)_2_ group was observed at 1365–1385 and 1167 cm^−1^; nevertheless, their intensities were weak. The occurrence of two medium intensity bands at 1083 and 1016 cm^−1^ may also suggest the presence of oxygenated moieties on the polymer surface resulting from the side reaction of oxidation of double bonds [45,46].

In the case of the oil-based materials (Figure 3b), besides the DVB characteristic bands, all the spectra also included those of ester moieties. They were observed as one separated band at 1744 cm^−1^ caused by stretching of the C=O bond (in esters), and another band due to C–O stretching in the range of 1250–1006 cm^−1^. The latter is more complex because it arises as a combination of several vibrations. The most characteristic were those of symmetric and asymmetric C–O–C stretching, observed as an intense band at about 1095 cm^−1^ and a less intense one in the range 1250–1130 cm^−1^, respectively. There was also a band at about 1020 cm^−1^ in the discussed region. However, this could not be unequivocally attributed to one characteristic vibration. Its origin was probably an effect of overlapping not only C–O ester vibration but also ether, or to a lesser extent, alcohol, which can be formed as side reaction products.

It is worth noting that a slight broadening of the carbonyl band of esters from the lower wavenumber values (up to 1680 cm^−1^), and a small deviation from the baseline of the spectra in the range of 3500–3200 cm^−1^, may indicate partial hydrolysis of some ester bonds and formation of intramolecular hydrogen-bonded carboxylic acid. This suggestion was confirmed by acid values obtained in the analytical determination.

Methylene group (–CH_2_–) is another characteristic structural fragment of oil-based materials. The presence of long-chain aliphatic carboxylic acids, which together with glycerol build triglycerides, contributes to an increase in the intensity of the methylene characteristic bands. In particular, this effect was visible for asymmetric and symmetric stretching vibrations at 2923 and 2854 cm^−1^, as well as deformation and scissoring ones at about 1460 cm^−1^. Slight differences in spectrograms result from the individual composition of the vegetable oils used in the syntheses [15,39,47,48,49].

The disappearance of the bands at about 3010 and 1654 cm^−1^ originated from vibrations of –CH=CH– (cis), which led to the conclusion that these bonds took part in the polymerization reaction, contributing to a permanent connection of these structural elements.

Some additional insight into the chemical properties of the synthesized materials was provided by an analysis of characteristic properties such as acid values and iodine values. Table 3 presents the values obtained for the prepared materials.

The acid value indicates the presence of free carboxylic acids in the polymer sample. Polymers C-DVB, L-DVB, and P-DVB carboxylic species can arise as an oxidation product of methyl groups or some C=C bonds, and in the case of citral derivatives, also by oxidation of aldehyde groups. Such processes are undesired side reactions. Slightly higher results of acid values were obtained for the oil-based polymers, which were a consequence of the slow hydrolysis of fats heated in the aqueous solution during synthesis. The course of such a side reaction was also proved by the above-described broadening of the characteristic band of the ester group.

The iodine value is informative of the availability of residual C=C in the polymer network. During the determination, bromine, and iodine contained in the Hanus reagent yielded quantitative addition to unsaturated bonds in the polymer network. The obtained results were within the range of 10–32 mgI_2_ per 100 g of a polymer sample, which is much lower than for vegetable oils (Table 1). Low values of IV indicate a high conversion of the reactants. However, non-zero values mean that some amounts of unsaturated bonds remained in the polymer structures. These could be equally unreacted double bonds of monomers or bonds that arose during the termination of polymerization, e.g., as a result of disproportionation reactions.

### 3.2. Analysis of Porous Structure

The analysis of porosity using low-temperature nitrogen adsorption-desorption measurements showed that the prepared polymers differed significantly in terms of their internal structure. Determined data regarding specific surface areas, total pore volume and mean diameter of pores are collated in Table 4. Citral and limonene-based polymers possessed very well-developed porous structures, while P-DVB was practically nonporous. In the case of oil-based materials, porosities were also developed; however, their values were not as high as those obtained for C-DVB or L-DVB.

**Figure 4:**
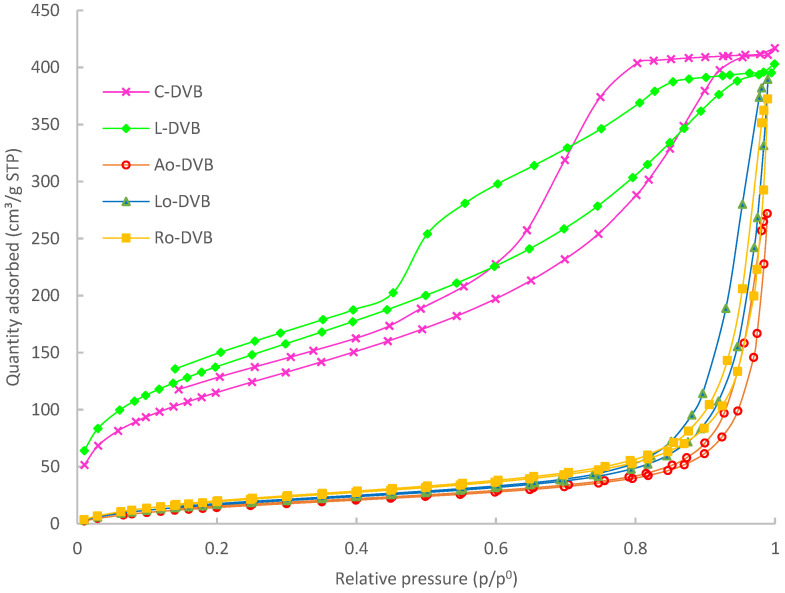
The nitrogen adsorption-desorption isotherms of the studied polymers.

Very interesting is the graph depicting the adsorption-desorption isotherms (Figure 4). It shows fundamental differences regarding the porous structure of the studied polymers. The isotherms of C-DVB and L-DVB can be classified as Type IV(a) according to the recently updated IUPAC classification [50].

A characteristic feature of this type of isotherm is the final saturation plateau, which in this case is very reduced, and the presence of the hysteresis loop, which proves that capillary condensation took place in the mesopores wider than a certain critical width (ca 4 nm). A small increase of adsorbed amount at p/p^0^ close to 1 can be due to the condensation of adsorbates in small macropores or inter-particle voids [50,51,52].

**Figure 5:**
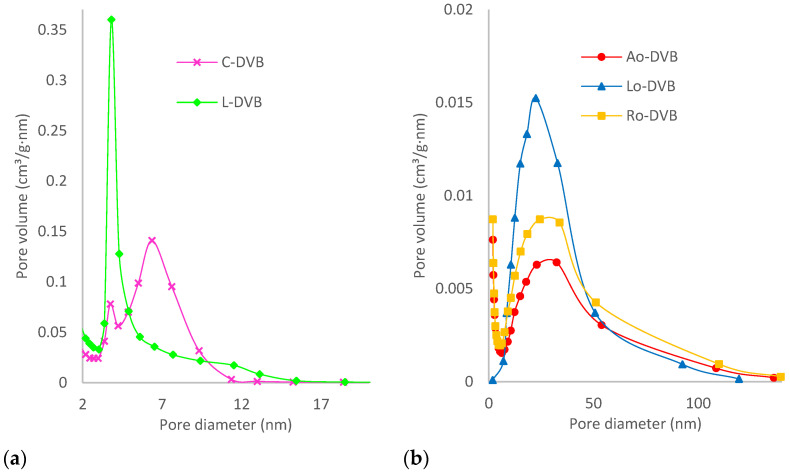
The pore size distribution plots (**a**) C-DVB and L-DVB, and (**b**) Ao-DVB, Lo-DVB, and Ro-DVB.

The hysteresis loop of C-DVB can be identified as H2(b). Such shape is characteristic of materials with complex porous structures in which network effects are important. Among them, pore-blocking mechanisms [50,51,52] and/or percolation effects [53,54] during desorption from pores of larger neck width size distribution are indicated as the most dominant. Therefore, the following interpretation can be made: in the porous structure of C-DVB dominant pores possess narrow cavity size distributions and wide neck size distributions. The characteristic (asymmetric) shape of the hysteresis loop was probably caused by the percolation effect and suggests an even more complex structure in which the pores are interconnected [53,54]. Figure 5a presents the pore size distribution (PSD) plots; the curve obtained for C-DVB was bimodal with two unsymmetrical peaks at about 3.75 and 6.36 nm. The latter was wider and larger. Although the curve was bimodal, the PDS remained within a narrow range of values from 2 to 12 nm.

For L-DVB, the hysteresis loop was more complex and probably should be considered as a combination of types H2(b) and H5 (Figure 4). The distinctive form of Type H5 resulted from the presence of both open and partially blocked mesopores in the polymer network. Another characteristic feature was the steep step down observed in the desorption branch, possibly as a consequence of cavitation [50,51,52,55]. Such an effect usually is located at p/p^0^ ca 0.4–0.5 when N_2_ at 77 K is used as adsorptive and was also found in the case of L-DVB. Therefore, considering the properties of the porous structure of the L-DVB polymer, it can be concluded that they were similar to C-DVB; the pores form an irregular network of cavities and channels connected to each other, but some were partially blocked. The PSD of L-DVB (Figure 5a) was a little broader than that of C-DVB. This was in the range of 2–15 nm; however, pores of 3–5.5 nm dominated the structure, making the porosity more uniform.

All the adsorption-desorption isotherms of the oil-based polymers were the same—Type V. This type of isotherm is observed for materials that poorly interact with adsorptive and when wetting of the surface by the adsorbate is not complete, especially at low relative pressure. At higher p/p^0^ values, stronger interaction between adsorbate molecules leads to the formation of clusters, then pore filling takes place accompanied by hysteresis. The shapes of these hysteresis loops resembled Type H3, however, and a characteristic steep step down was not observed, which proved that the cavitation process did not occur in the case of the oil-based polymers. This type of hysteresis is representative of non-rigid aggregates of plate-like particles or porous materials with macropores that are not completely filled with the adsorbates. Taking into account the chemical structure of the substrates used in the syntheses and considering the PSD plots (Figure 5b) of the prepared polymers, it should be noted that the studied materials combined both of these characteristic features. The polymeric network was built of non-rigid aliphatic chains of different lengths bound together with rigid plate-like aromatic rings (Figure 2) forming slit-shaped pores with walls not necessarily parallel. For this reason, the porous structure of these polymers had predominate mesopores with sizes 6–50 nm, but macropores with widths up to 140 nm were also present.

The simulation of adsorption isotherms for pores of different widths, lengths, and shapes performed by Fan et al. [56] demonstrated that when the pore width is greater than a certain critical size, evaporation takes place at a higher pressure than is needed for cavitation. As a result, the evaporation mechanism changes from cavitation to pore blocking and this explains the above-mentioned lack of typical cavitation steep step down on the desorption branch of the isotherms as well as the shifting of the hysteresis loop towards a higher value relative pressure (p/p^0^).

**Figure 6:**
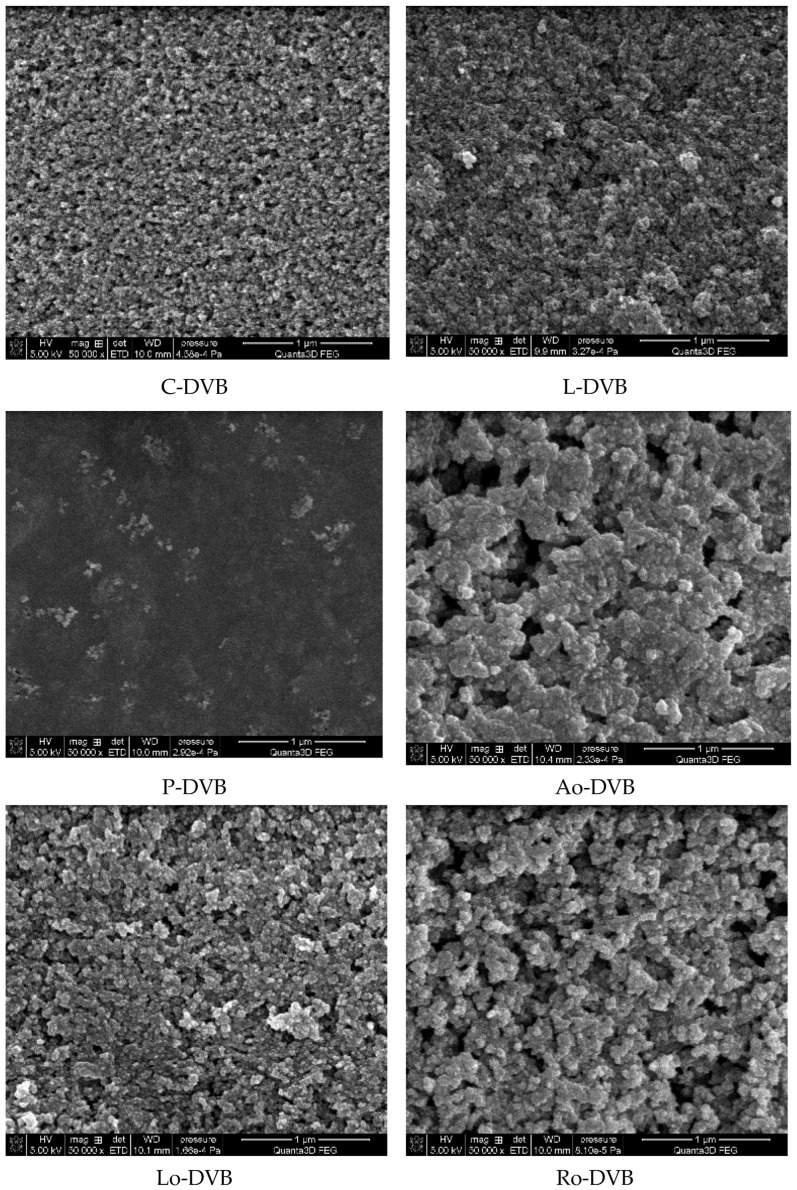
SEM images of the studied polymers.

Figure 6 shows SEM images depicting the morphology of the terpene-based and oil-based polymers. A porous structure formed by irregularly shaped cavities was clearly visible. Macropore entrances with sizes from 50 nm (C-DVB and DVB) to 500 nm (Ro-DVB and Ao-DVB) were observed. The only exception was the P-DVB polymer, whose surface was almost non-porous.

### 3.3. Thermogravimetric Analysis

**Figure 7:**
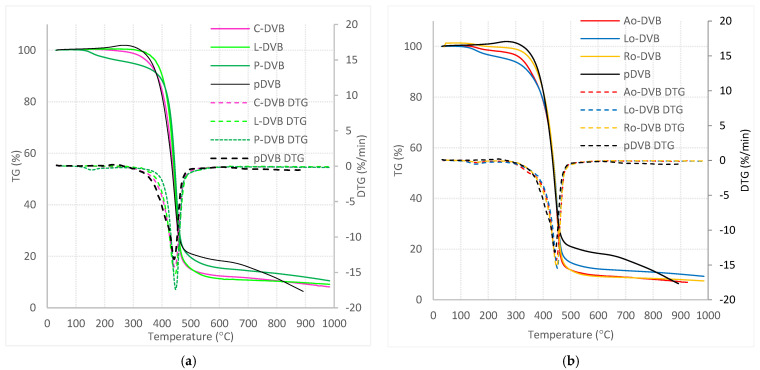
Thermogravimetric analysis results (**a**) C-DVB and L-DVB, and (**b**) Ao-DVB, Lo-DVB, and Ro-DVB.

In order to evaluate the thermal stability of the studied polymers, thermogravimetric analysis was conducted. Figure 7 and Table 5 collate the obtained data. The temperature at which the 1% weight loss of the polymer occurred (T_1%_) was taken as the initial decomposition temperature. Among the terpenes containing the polymers, the least thermally stable was P-DVB, whose decomposition began above 150 °C, which can be related to the partial oxidation of the polymer. Its decomposition ran in two stages that can be observed on the DTG plot as peaks at 155 and 447 °C (T_D1_ and T_max_). C-DVB and L-DVB were thermally stable up to ca. 300 °C and their decomposition was a one-stage process occurring in the range of 350–550 °C with the temperature of the maximum decomposition rate (T_max_) being 445 °C. The process proceeded rapidly and was accompanied by a loss of approximately 89% of the sample weight. Comparing temperatures of 1% and 5% mass loss of the samples (T_1%_ and T_5%_), the polymer L-DVB can be indicated as more thermally stable. For comparison, purpose curves for homopolymer pDVB (composed of only DVB under the same conditions as studied polymers), were added. As can be seen, decomposition of pDVB began at 200 °C with the oxidation process and progressed up to 480 °C. This meant that the C-DVB and L-DVB had better thermal stability than the pDVB.

The thermal properties of the oil-based polymers were similar to those of the P-DVB. In this case, decompositions also exhibited two stages: again the first stage was slow and the other was rapid. The temperature ranges in which the discussed processes took place were also almost the same, 153–161 °C and 447–451 °C for T_D1_ and T_max_, respectively. However, the range of decomposition temperatures shifted downwards by around 50 degrees. The mass loss of the samples varied from 83 to 90%. Among the oil-based polymers, the most thermally stable was obtained with the use of rapeseed oil. The T_1%_ and T_5%_ values for the Ro-DVB polymer were comparable to those for C-DVB.

The studies of the thermal properties of the synthesized polymer materials allow them to divide into two groups with different thermal stability. The first one included materials with lower temperature resistance (P-DVB, Lo-DVB, and Ao-DVB) than pDVB, for which the recommended operating temperature should not exceed 100 °C, while the second one included C-DVB, L-DVB, and Ro-DVB, which can be used even at elevated temperatures up to 270–300 °C.

### 3.4. Sorption Abilities Tests

Sorption tests were carried out in dynamic mode. For the sorption experiments C-DVB, L-DVB, Lo-DVB, and Ro-DVB, polymers were chosen as representatives of each group possessing the highest porous structure parameters.

#### 3.4.1. The Sorption of the Phenols Mixture

**Figure 8:**
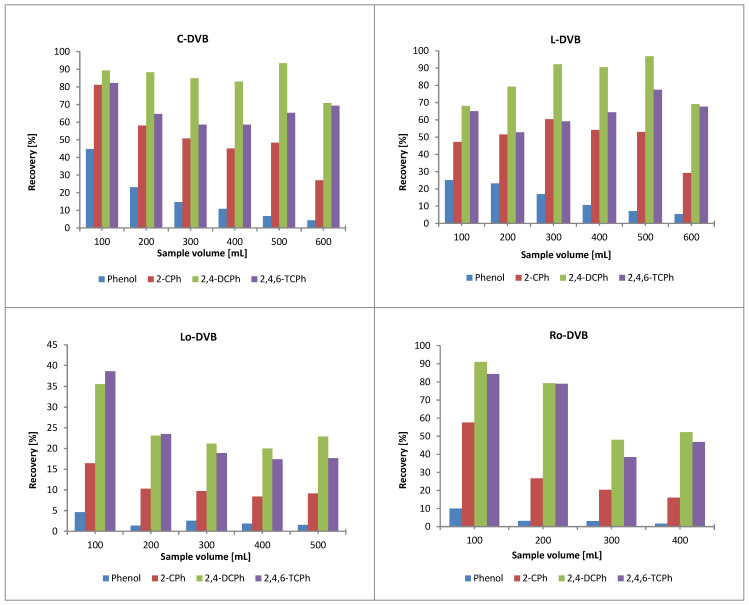

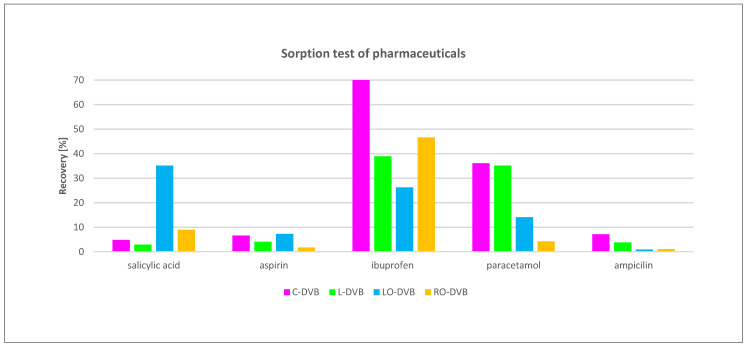
SPE results for C-DVB, L-DVB, Lo-DVB, and Ro-DVB.

The upper panels of Figure 8 present the results of the sorption of the phenolic compounds on the above specified materials.

In the first pair of polymers, 2,4-DChP showed the highest affinity to the C-DVB and L-DVB surfaces. This compound also achieved the highest recoveries even for sample volumes as large as 500 mL. A slight increase in recovery in this range of sample volumes may be caused by the longer interaction time of the sorbent with the solution. At a volume of 600 mL, a decrease in the recovery value was observed; thus, a breakthrough volume for this compound had been reached. 2-ChP and 2,4,6-TChP initially showed similar recoveries of about 50–80% for small sample volumes, but as the sample volume increased, the recoveries for the former compound gradually decreased and for the latter, they increased. This phenomenon can be explained in the following way: as the volume of the sample increased, the interaction time of the sorbent with the solution became longer, and this in turn may have led to a situation in which the previously adsorbed 2-ChP was replaced by 2,4,6-TChP, which interacted more strongly with the polymer surface. The process of sorption of aromatic compounds at the polymer-solution interface showed some analogies to the extraction process in the octanol-water system; therefore, the factors of lipophilic and hydrophilic properties, such as the octanol-water partition coefficient and water solubility, may be useful for explaining some dependencies related to the course of processes that took place during the solid phase extraction [57]. Comparing the logP and s_water_ data (Table 6) allowed for an explanation of why the molecules of 2-ChP could be replaced by those of 2,4,6-TChP. The higher logP value and lower solubility in water of 2,4,6-trichlorophenol indicated that it was more lipophilic than 2-ChP; therefore, its interaction with the polymer surface was stronger. On the other hand, the recoveries of 2,4,6-TChP were up to 25% lower than those obtained for 2,4-DChP. Taken together, the logP values for the two compounds suggested that 2,4,6-TChP should be adsorbed better than 2,4-DChP, but the obtained results showed an inverse relationship. In order to understand why this was so, the values of the acid dissociation constants pKa (Table 6) should be taken into account. Under the experimental conditions, the tested solutions had a slightly acid to neutral pH. In such an environment, the pKa of 2,4,6-TChP was close to the pH value, and therefore, dissociated molecules were also present in the solution. Excluding the possibility of electrostatic interactions, their interactions with the sorbent were weaker than these of the undissolved molecules, which in turn reduced the efficiency of the sorption process. Phenol is the compound with the lowest affinity for both polymers. The maximum recoveries of this compound were 40 and 20% for C-DVB and L-DVB, respectively, and they decreased gradually with increasing sample volume. This was a consequence of good water solubility and the relatively low lipophilic character of phenol.

In the case of the second pair of sorbents (Lo-DVB and Ro-DVB), the observed relationships were almost the same as those for the terpene-based polymers. However, due to the oil-based polymers having around five times lower specific surface areas, the effectiveness of these materials was slightly lower. Additionally, attention should be paid to the PSD of the discussed materials. This pair of polymers had a much larger proportion of wider mesopores and macropores in their structure, and narrow-width pores made up a small fraction of the total pore volume, especially in the case of Lo-DVB. For this reason, although both polymers possessed quite similar values of the porous structure parameters, higher recoveries were obtained for Ro-DVB, which had a greater portion of pores with widths below 6 nm than the polymer Lo-DVB.

**Figure 9:**
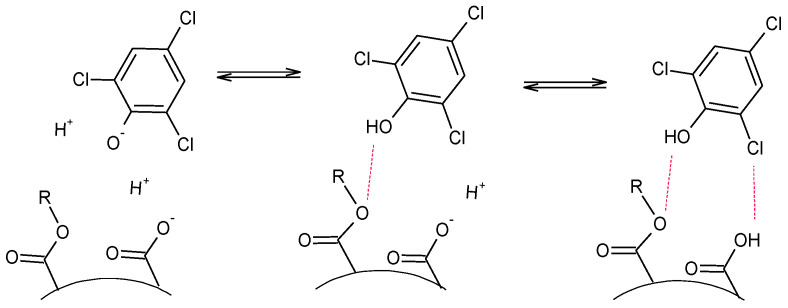
Scheme of the acid-base equilibria at the oil-based polymer and the phenols solution interface.

It is noteworthy that for Lo-DVB and Ro-DVB sorbents, the difference in the affinity of 2-ChP and 2,4,6-TChP to their surface was much more strongly pronounced. For most of the results, the recoveries for 2-ChP were approximately 50% lower than those for 2,4,6-TChP. However, this was not a result of the reduced sorption of 2-ChP on these polymers, but an increased affinity of 2,4,6-TChP towards their surfaces. This became more clear when the recovery values of 2,4,6-TChP with 2,4-DChP are compared. In this series of studies, they were on a similar or slightly lower level. To elucidate this observation, attention should also be paid to the chemical properties of the studied polymers. One of the factors that describe the acid properties of polymers is the acid value, which is shown in Table 3 and discussed in Section 3.1. The AVs for the terpene polymers were low, while those for the oil-based polymers were significantly higher. If a polymer having acidic groups is in a solution, such moieties will affect its acid-base balance. Even a slight increase in the concentration of hydrogen ions in the solution shifts the state of the dissociation equilibrium of 2,4,6-TChP towards the undissociated form, which is adsorbed to a greater extent than the ionic form. This is reflected by the presented results and schematically shown in Figure 9. For simplicity, the figure shows only 2,4,6-TChP molecules (with the lowest pKa value among the phenols), for which these processes are of the greatest importance in explaining the obtained results. As shown in Figure 9, the sorption process of the chlorophenols was supported by the possibility of the formation of hydrogen bonds between the substituents in the phenol aromatic ring and the surface functional groups of the polymer.

However, the most important role in this uptake process was played by the Van der Waals interactions as well as the π electron interactions between the aromatic rings of the sorptives and the rings of the polymer cross-linker (divinylbenzene).

#### 3.4.2. The Pharmaceuticals Sorption

The above considerations also justified the obtained results of drug sorption on the tested polymers. Salicylic acid and aspirin are acidic compounds (pKa in the range 2.8–3.5) that are well soluble in water. Their recoveries do not exceed 10% because in their aqueous solution under the conditions of the experiment, the dominant forms were the dissociated ones. For salicylic acid, the highest recovery value (ca. 35%) was recorded for Lo-DVB, i.e., the polymer with the most acidic surface. The ionized salicylic acid in contact with the strongly acidic surface of the Lo-DVB polymer began to turn into undissociated species, the sorption of which was more effective. This description was in line with the previous one regarding the sorption of 2,4,6-TChP.

Aspirin, which is an acetyl derivative of salicylic acid, is slightly more soluble in water and has a less acidic character, which justified the lower recoveries and a less pronounced influence of the acidity of the polymer surface on the obtained recovery results. A similar observation was also reported by Grochowicz et al. [34].

Ampicillin in its structure, apart from the acidic carboxyl group, also possessed amine and amide groups, which gave it a basic character. The polarized nature of these chemical bonds caused the hydrophilic character of the ampicillin to be predominant, which was confirmed by one of the lowest log Ps and its good solubility in water. Moreover, its large molecule was the most complex in terms of spatial structure compared to the other test compounds used in this study. All these features undoubtedly influenced its interaction with the polymers.

Among the selected pharmaceuticals, ibuprofen and paracetamol had the highest affinity to the tested polymers. For ibuprofen and paracetamol, the recoveries were in the range of 27–70% and 5–40%, respectively.

**Figure 10:**
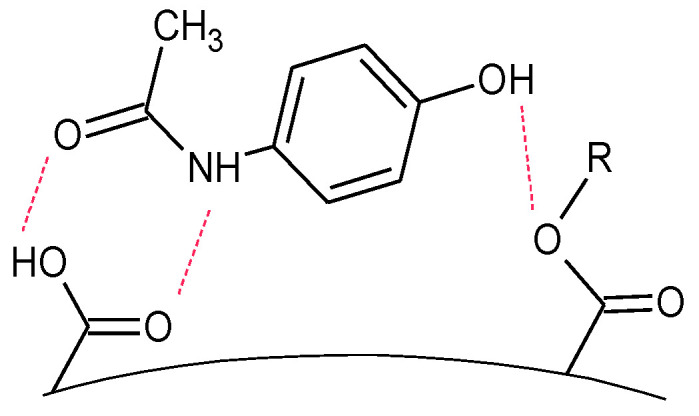
Scheme of possible interactions between a paracetamol molecule and an acid functional group on the polymer surface.

When analyzing the results obtained for paracetamol, it should be stated that higher values of recovery were obtained for terpene-based polymers with highly developed porosity and were much lower for the oil-based ones with much lower specific surfaces. The Lo-DVB and Ro-DVB polymers had similar porous structure parameters but the recovery for the former, which surface was twice richer in acid functional groups, was three times higher than that for the latter. Paracetamol in its structure had two functional groups attached to the aromatic ring: a hydroxyl and an amide. There were strongly negative electrostatic potentials accumulated in the O–H and C=O groups, while in the NH group, a positive potential was localized [30]. The presence of these oxygen functionalities gave this molecule a hydrophilic character (logP, s_water_), which resulted in its lower affinity towards the polymers. On the other hand, the presence of a partial positive charge on the paracetamol molecule favored its stronger interaction with acidic surfaces, as is schematically shown in Figure 10. This effect is clearly visible when comparing the Lo-DVB and Ro-DVB recoveries.

The values of paracetamol recoveries for terpene-based polymers differed very little. However, it should be noted that this difference was the result of two independent factors—surface porosity and acidity. Namely, C-DVB had a smaller specific surface area, but about twice more acid groups than L-DVB with a larger surface area. The paracetamol OH and CONH groups formed associations with the functionalities of the polymer using the other electrostatic potentials accumulated around these moieties. The specific interactions of paracetamol with more numerous acidic groups of C-DVB, compensated for the loss of uptake due to the lower, by about 100 m^2^/g, specific surface area. For this reason, it was finally observed that the sorption effect was almost identical for both materials. Summing up the considerations regarding paracetamol sorption on the polymers under study, it should be emphasized that the mechanism of the process should take into account not only the Van der Waals and π-interactions but also the possibility of donor-acceptor or hydrogen bond formation.

The ibuprofen recoveries on the studied polymers reached the highest values among all the tested pharmaceuticals. Similarly to the previously discussed adsorbates, for ibuprofen, the influence of the polymer’s porosity development on the obtained recovery values was also the most pronounced. For the terpene-based polymers, the positive effect of the presence of surface acidic functionalities should also be mentioned because the recoveries for C-DVB were almost twice as much as that for L-DVB. However, such a relationship was not observed for the pair of oil-based polymers, both for ibuprofen and chlorophenols. Recoveries for Lo-DVB in all cases were ca 50% lower than for the other considered polymers. A comparative analysis of all the collected data allowed for the identification of some analogies to the recovery values obtained for the 100 mL samples of 2,4,6-TChP or 2,4-DChP. Specifically, 2,4,6-trichlorophenol had chemical properties (log P, s water, and to a lesser extent pKa) similar to those of ibuprofen. This suggests that the ibuprofen sorption process might follow the same mechanism as for chlorophenols.

The presence of an aromatic ring and alkyl or halogen substituents in the molecules of these adsorbates caused by the uptake on the polymer surface was based on not very strong physical interactions including hydrophobic, Van der Waals, and aromatic π-electron interactions. However, the presence of OH or COOH groups with a strong negative potential in the molecule additionally led to specific interactions, such as hydrogen bonding and the formation of the donor-acceptor complexes, which contributed to the sorption process, favoring or countering it, depending on the properties of the environment and the adsorbent. Therefore, when comparing the sorption of the same compound on different polymers, the reasons for the different sorption results should be sought in the structure and surface chemistry of these materials.

**Figure 11:**
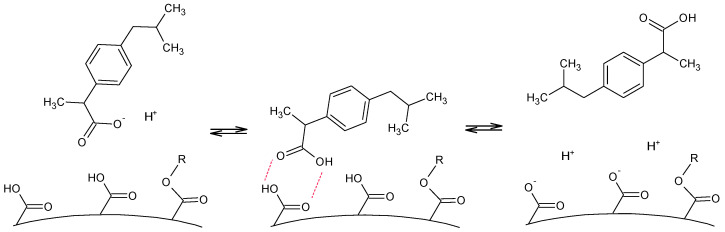
Scheme of possible acid-base balance between an ibuprofen molecule and acid moieties of the polymer.

The highest value of AV for Lo-DVB indicated that the concentration of acidic functional groups on its surface was high. This dissociation of mainly carboxylic moieties not only changed the acid-base balance of the solution but also left the polymer surface negatively charged, which was not beneficial for the sorption of molecules with a negative potential accumulated around their carboxyl, hydroxyl, or halogen groups. Such repulsive electrostatic interactions tended to move away or repel the negatively charged side of the molecule from the polymer surface, which in turn reduced the interaction of the adsorbate with the sorbent; this is schematically presented in Figure 11 and in the form of a simple animation submitted as a Supplementary File (Video S1).

Table 7 shows the comparative recovery results of drugs that were obtained for different types of sorbents. It can be seen that the data varied greatly and depended both on the type of sorbent and the elution conditions. Higher results were obtained for sorbents modified with specific functional groups, the presence of which enhanced the interactions with drug moieties; in other cases, the results were lower.

Taking into account the results presented in this work it should be stated that ibuprofen recovery for C-DVB is comparable and the recovery of salicylic acid is even higher than those reported in the literature.

The sorption ability tests presented above showed that the prepared bio-based polymers containing natural origin components in their structure can be used to effectively remove selected phenolic and pharmaceutical compounds from aqueous solutions. The analysis of the influence of various factors on the obtained results allowed indicates that more appropriate adjustment conditions of the process (e.g., pH) would achieve even higher recoveries. Moreover, it should be emphasized that these polymers can be regenerated and reused even from large volumes of water samples while maintaining high desorption efficiency.

## 4. Conclusions

This work presents a method of synthesis of bio-based porous polymer microspheres with the use of terpene compounds and vegetable oils as substrates. The obtained polymers were characterized using chemical and instrumental methods, which allowed the structural and chemical differences of these materials to be determined.

The citral- and limonene-based polymers possessed well-developed porous structures (S_BET_ 428 and 509 m^2^/g) with more uniform pores compared to the oil-based materials, with pores of a wider range of diameters and specific surface areas not exceeding 100 m^2^/g. Furthermore, the thermal resistance of the polymers was diversified. P-DVB, Lo-DVB, and Ao-DVB were less thermally stable and should not be utilized above 100 °C, while C-DVB, L-DVB, and Ro-DVB can be safely employed even up to 270–300 °C. Chemical analysis proved the presence of acidic groups on the surface of the studied polymers. The oil-based materials possessed about 21–44 mg/g of free carboxylic groups and the terpene-based ones possessed only 4–9 mg/g.

This surface acidity together with the porosity turned out to have a particularly significant influence on the sorption process of selected test compounds. The presence of functional groups capable of forming hydrogen bonds or acid-base complexes with the adsorbates significantly affected the uptake from the solution, and consequently, the recovery of adsorbed compounds. However, for a comprehensive explanation of the obtained results, it was also necessary to take into account the properties of adsorbates and the processes that may occur in the solution and at the solution-polymer interface.

The highest sorption efficiency towards dichlorophenol and trichlorophenol was achieved by C-DVB and L-DVB; the recoveries for these phenols were in the range of 70–100% and 60–80%, respectively. Results at this level were maintained even for 500 mL samples in the fifth concentration cycle. The efficiency of Ro-DVB was lower due to the less developed porosity. Although, the recoveries for di- and trichlorophenol were 80–90% and 80–85%, respectively, they were only valid for the 100 and 200 mL samples. The lowest recovery results (3–45%) were obtained for phenol because of its hydrophilic nature and good solubility in water. All the presented polymers were very efficient in the removal of ibuprofen (25–70%) and somewhat less effective in the removal of paracetamol (5–37%). A 35% recovery of salicylic acid was reached only by the LO-DVB polymer.

The undoubted advantage of the presented bio-based polymers is their good regeneration potential and the possibility of reuse without losing the recovery efficiency.

## Figures and Tables

**Table 1:** Mean values of fatty acid composition in oils.

Type of Oil	Saturated	Monounsaturated	Polyunsaturated		Average Number of C=C	Iodine Value (mg I2/100 g)	Ref.
	16:0 and 18:0 (%)	18:1 n-9 (%)	18:2 n-6 (%)	18:3 n-3 (%)			
Argan	18.5	46	35	0.5		91–110	[9]
Linseed	10	20	15	55	6.6	168–204	[10]
Rapeseed	7	63	20	10	3.8	90–120	[10]

**Table 2:** Designations of the prepared polymers.

Polymer	Acronim
Citral-divinylbenzene	C-DVB
Limonene-divinylbenzene	L-DVB
Pinene-divinylbenzene	P-DVB
Argan oil- divinylbenzene	Ao-DVB
Linseed oil-divinylbenzene	Lo-DVB
Rapeseed oil-divinylbenzene	Ro-DVB

**Table 3:** Characteristic chemical parameters: acid values (AV) and iodine values (IV).

Polymer	AV (mg KOH/g)	IV (mg I_2_/100 g)
C-DVB	9.26	19.61
L-DVB	4.12	31.59
P-DVB	7.21	10.04
Ao-DVB	21.24	17.00
Lo-DVB	43.90	19.77
Ro-DVB	22.86	20.45

**Table 4:** Porous structure parameters of the prepared polymeric materials.

Polymer	S_BET_ (m^2^/g)	V_tot_ (cm^3^/g)	D_BJH_ (nm)
C-DVB	428	0.645	5.5
L-DVB	509	0.624	4.6
P-DVB	-	-	-
Ao-DVB	64	0.421	24.7
Lo-DVB	72	0.603	26.3
Ro-DVB	84	0.576	25.4

**Table 5:** Results of thermogravimetric analyses.

Polymer	T_1%_ (°C)	T_5%_ (°C)	T_D1_ (°C)	T_max_ (°C)	T_D range_ (°C)
C-DVB	294	355	-	445	350–550
L-DVB	345	381	-	445	350–550
P-DVB	154	296	155	447	350–550
Ao-DVB	175	321	161	447	300–500
Lo-DVB	144	269	153	451	300–500
Ro-DVB	298	353	160	449	300–500
pDVB	200	365	-	443	200–480

**Table 6:** Properties of the adsorbates [57].

Compound	Phenol	2-ChP	2,4-DChP	2,4,6-TChP	Salicylic Acid	Aspirin	Ibuprofen	Paracetamol	Ampicillin
logP	1.46	2.15	3.06	3.69	2.26	1.19	3.97	0.46	0.93
s_water_ [g∙L^−1^]	84	28.5	4.5	0.85	2	2.5 ^a^	0.021 ^b^	14	50
pK_a_	9.99	8.56	7.89	6.23	2.98; 13.6 ^c^	3.49	4.45	9.38	2.8; 8.42 ^d^

logP—octanol-water partition coefficient, s_water_–solubility in water determined at 20 °C or ^a^ (at 15 °C) or ^b^ (at 25 °C), pK_a_—logarithmic acid dissociation constant value determined at 25 °C or ^c^ (at 20 °C) or ^d^ (for acid form at 23 °C).

**Table 7:** Comparison table of drug recoveries reported in the literature.

Sorbent Type	Drug	Matrix	Eluent	% of Recovery	Ref.
pDVB pDVB-SO_3_H	Salicylic acid	water	MeOH	4.6 6.6	[38]
SAPA-MAA (SiO_2_-Al_2_O_3_-Propyl amine-Methyl methactrylate)	Salicylic acid	water	H_2_O and EtOH 0.01 mol/L NaOH and 35% EtOH	<12.5 ~86	[58]
pDVB DVB-GMA(75-25)-H_2_SO_4_	Aspirin	water	MeOH	35 17	[36]
Poly(4VP-14DVB Poly(4VP-TRIM)	Ibuprofen	water	MeOH	~70 ~40	[34]
pDVB pDVB-SO_3_H	ibuprofen	water	MeOH	34.8 16.2	[38]
aqueous biphasic systems (ABS)	Paracetamol	Pharmaceutical waste	water	80–100	[59]
VBC-DVB poly(chloromethylstyrene-co- divinylbenzene (PVBC-DVB)	Ampicillin	acetonitrile 0.1 mol/L aqueous NaOH (9:1, *v*/*v*)	methanol: glacial acetic acid (8:2, *v*/*v*)	~80	[60]
amino-functionalized silica gels covered with MAA—EGDMA	Ampicillin	Milk blood	methanol–acetic acid (4:1, *v*/*v*). The	64–82 67–78	[61]

## Data Availability

The data presented in this article is available on request from the corresponding author.

## Supplementary Materials

The following supporting information can be downloaded at: https://www.mdpi.com/article/10.3390/polym14245389/s1, Video S1: influence of polymer surface acidity on ibuprofen sorption.

## Abbreviations

AIBN	α,α’-Azoiso-bis-butyronitrile
Ao	argan oil
Ao-DVB	argan oil and DVB copolymer
ATR/FTIR	attenuated total reflectance mode infrared spectroscopy with Fourier transform
AV	acid value
C	citral
C-DVB	citral and DVB copolymer
D_BJH_	average pore diameter according to the Barrett–Joyner–Halenda model
DVB	divinylbenzene
HPLC	High-performance liquid chromatography
IV	iodine value
L	limonene
L-DVB	limonene and DVB copolymer
Lo	linseed oil
Lo-DVB	linseed oil and DVB copolymer
logP	octanol-water partition coefficient
MeOH	methanol
P	pinene
P-DVB	pinene and DVB copolymer
pKa	logarithmic acid dissociation constant
PSD	pore size distribution
PVAL	poly(vinyl alcohol)
Ro	rapeseed oil
Ro-DVB	rapeseed oil and DVB copolymer
S_BET_	specific surface area according to the standard Brunauer–Emmett–Teller theory
SPE	dynamic solid phase extraction method
s_water_	solubility in water
T_1%_,T_5%_	the temperature at which the 1% or 5% weight of the sample is lost
T_D_ and T_D1_	decomposition temperature and first-stage decomposition temperature
TG/DTG	thermogravimetry/differential thermogravimetry
Tmax	temperature of the maximum decomposition rate
UV	ultraviolet–visible
V_tot_	total pore volume
2-ChP	2-chlorophenol
2,4-DChP	2,4-dichlorophenol
2,4,6-TChP	2,4,6-trichlorophenol

## Appendix A

**Determination of the acid value (AV)** for highly cross-linked porous polymers by the direct (standard) method may be subjected to a large error due to the relatively slow process of penetration of the titrant through the porous polymeric network. In order to ensure a sufficiently long time for diffusion and ion exchange, the determination of the acid number was performed using a back titration method as follows: the polymeric sample (100 mg) was weight into Erlenmeyer flask and 1 mL of methanol was added to improve the sample wettability, next 10 mL of KOH aqueous solution (0.081 M) was measured. Sample was mixed, corked and left at room temperature for about 60 min. Under these condition, only acidic species are able to react with diluted alkali solution. After this time, the excess of unreacted KOH was titrated with a standard HCl solution (0.05 M) at the presence of an indicator – methanol solution of methyl red and methylene blue. A blank test was carried out in the same way. The acid value was calculated according to an Equation (A1).

(A1) $$AV=56.1\cdot C_{HCl}\cdot \frac{B-V}{w}$$

where: AV–acid value (mg KOH/g), 56.1–molecular weight of KOH, C_HCl_–molar concentration of titrant solution (mol/L), B–volume of titrant (mL) consumed for blank titration, V–volume of titrant (mL) consumed by the sample at the equivalent point, w–weight of the sample (g).

To determine **iodine value (IV)** polymeric samples (200 mg) were suspended in 10 mL of chloroform and 15 mL of Hanus reagent (4.0 g bromine, 6.5 g iodine 500 mL of conc. acetic acid) was added. Next, samples were left in the dark place for 30 min. to allow the reaction between the iodine and the unsaturated bonds of the polymer. After this time 50mL of distilled water and 15 mL of 10% aqueous KI solution was poured and the resulting mixture was titrated with standard Na_2_S_2_O_3_ solution (0.179 M) until the contents of the flask turn orange. Then 1 ml of 1% starch solution as an indicator was added and the titration was continued up to the time of starch was completely discolored. The blank test was performed in the same way. To calculate IV following equation was used:

(A2) $$IV=126.9\cdot C_{Na_{2}S_{2}O_{3}}\cdot \frac{1}{10}\cdot \frac{B-V}{w}$$

where: IV–iodine value (mg I_2_/100 g), 126.9–molecular weight of I_2_, C_Na2S2O3_–molar concentration of titrant solution (mol/L), 1/10–is conversion factor (taking into account the conversion of milliliters per liter and the weight of the sample per 100 g) B–volume of titrant (mL) consumed for blank titration, V–volume of titrant (mL) consumed by the sample, w–weight of the sample (g).

**Table A1:** Wave numbers of monomers.

C	L	P	Ao	Lo	Ro	DVB
2968.457	3083.453	3024.865	3007.353	3468.442	3006.422	3087.813
2915.508	3011.003	2984.971	2923.037	3009.001	2923.236	3055.264
2857.437	2965.117	2916.507	2853.451	2924.598	2853.53	3007.276
2759.877	2917.433	2879.387	1744.187	2854.047	1744.006	2966.002
2728.047	2856.229	2834.379	1463.291	1742.008	1459.333	2931.469
1672.158	2834.492	1658.384	1418.141	1458.949	1418.186	2872.61
1631.983	1644.483	1469.331	1398.631	1417.89	1376.787	1818.536
1611.446	1436.203	1445.45	1377.167	1376.043	1235.209	1630.315
1442.202	1375.915	1380.498	1317.384	1232.913	1161.082	1595.062
1377.41	1241.308	1365.077	1235.387	1162.476	1118.995	1575.97
1193.912	1198.81	1329.001	1160.792	1098.037	1096.403	1509.782
1153.376	1154.724	1264.869	1119.149	1031.138	1030.789	1484.909
1120.527	1051.365	1218.838	1097.268	972.9274	987.3027	1453.605
1043.012	1015.976	1204.177	1032.626	913.4726	913.4422	1442.674
983.6472	956.5505	1165.589	965.4592	913.4726	871.3782	1400.093
841.8421	914.0626	1125.272	913.2107	722.6462	722.4664	1281.776
821.108	886.0672	1084.328	722.1391			1166.228
749.2379	797.4017	1062.569				1118.94
	758.8232	1014.694	0			1092.561
		952.9739	0			1027.662
		927.7273	0			1013.46
		886.3422	0			988.2506
		856.524	0			904.0132
		840.8879	0			844.4089
		786.8981	0			800.1335
		771.23	0			707.5775
		669.0419	0			669.8808
		619.1145	0			617.703
